# Completeness of repeated patient-reported outcome measures in adult rehabilitation: a randomized controlled trial in a diverse clinical population

**DOI:** 10.1186/s12913-024-12103-8

**Published:** 2024-12-24

**Authors:** Anne-Lene Sand-Svartrud, Ingvild Kjeken, Renate Foss Skardal, Gunhild M. Gjerset, Tonje Jossie Johnsen, Anne Dorte Lyken, Hanne Dagfinrud, Rikke Helene Moe

**Affiliations:** 1 Health Services Research and Innovation Unit, and Center for Treatment of Rheumatic and Musculoskeletal Diseases (REMEDY), Diakonhjemmet Hospital, Oslo, Norway; 2 Sørlandet Rehabilitation Center, Eiken, Norway; 3 Montebello Rehabilitation Center, Mesnali, Norway; 4 Hernes Occupational Rehabilitation Center, Hernes, Norway

**Keywords:** Rehabilitation, Data quality, Completeness, Patient-reported outcome, Patient-reported outcome measures

## Abstract

**Background:**

Data collection through patient-reported outcome measures (PROMs) is essential for the purpose of rehabilitation research and registries. Existing problems with incomplete PROM data may relate to the patient burden and data set length. This study aimed to analyse response patterns and degree of data completeness in systematic outcome assessments conducted within a clinical study in a multidisciplinary rehabilitation setting, comparing completeness of a brief and a longer set of PROMs.

**Methods:**

The Norwegian RehabNytte Cohort was developed to monitor patients’ long-term benefit of rehabilitation and progress on PROMs. Adults admitted to one of 17 institutions providing mostly inpatient rehabilitation in secondary healthcare were included between January 2019 and March 2020, and followed for one year. For the purpose of the current randomized controlled trial, the Cohort-patients in 16/17 institutions were randomized to complete either a brief or a longer set of PROMs at admission, discharge, and after 3, 6 and 12 months. The brief set comprised the EQ-5D-5L and additional generic PROMs commonly used in rehabilitation settings. The longer data set expanded upon the brief set by including the Patient-Specific Functional Scale and the 29-item version of the PROMIS Profile instruments. Completeness was measured as the extent of present applicable PROM data at each time point. In addition, we assessed response patterns in terms of dropout rates and intermittently missed assessment events. The RehabNytte study is registered under ClinicalTrial.gov (NCT03764982, first posted 05.12.2018).

**Results:**

Of the 2904 patients included, 1455 were assigned to the brief data set and 1449 to the longer data set. The majority of patients were referred to rehabilitation for rheumatic and musculoskeletal diseases (39.3%) or cancer (26.9%). The data set completeness was significantly higher in the brief set compared to the longer (*p* < 0.001). From admission to 12 months follow-up, differences in completeness between groups ranged from 6.5 to 12.6 percentage points, consistently favouring the brief set. Completeness was highest at admission, reaching 96.8% (95% CI 0.96–0.98) for the brief set and 84.2% (95% CI 0.82–0.86) for the longer set. The lowest completeness was observed at discharge, with 46.0% (95% CI 0.43–0.49) for the brief set and 39.5% (95% CI 0.37–0.42) for the longer one. Discharge was the only time point without automatic reminders to non-responders from the digital data collection system. Patients responding to the longer data set exhibited the highest dropout rates, while degree of intermittent missing data was comparable between groups. In both groups, only one-third of patients provided complete or partly responses at all five time points.

**Conclusions:**

This study demonstrated that a brief set of PROMs achieved higher data completeness compared to a longer set, when used for repeated measurements in a rehabilitation research setting.

**Supplementary Information:**

The online version contains supplementary material available at 10.1186/s12913-024-12103-8.

## Background

Monitoring patient-reported outcome measures (PROMs) is vital in rehabilitation research and registries, providing insights into the patients’ perspectives on health status and the benefits of rehabilitation interventions [1, 2]. A longitudinal approach, utilizing PROMs through repeated measurements, is particularly emphasized for capturing the long-term progress or management of non-communicable health conditions [3–6]. Aggregated PROM data can inform decision-making for diverse stakeholders, including researchers, rehabilitation managers, and public authorities, by shaping healthcare policies, guiding resource allocation, and driving quality improvements at local, regional, and national levels [5, 7, 8]. Optimal use of PROM data requires a high response rate from patients and careful selection of appropriate instruments by the stakeholders [9–13].

Generic PROMs, capturing a wide spectrum of health domains, are applicable in the context of disease heterogeneity and complex health experiences often encountered in rehabilitation settings. Important health domains include symptoms, functional status, daily activities, social and vocational participation, goal setting/attainment, emotional wellbeing, and quality of life [13–15]. While a package of different questionnaires may be of relevance for patients, providers, researchers, and regulatory authorities, the individual burden associated with completing a long set of PROMs must be carefully considered [7, 13]. By using digital data collection, completeness of PROM data might improve compared with that attained with paper-based methods [16, 17]. However, data collection using PROMs is still affected by missing or incomplete data, implicating loss of statistical power and misleading conclusions on progress and outcomes in research, health policy, and clinical practice [17, 18].

Efforts to assess and improve completeness of data are recommended steps towards high-quality data sets that are suited to serve its purpose [19]. In this paper, we refer to completeness as the extent to which applicable data are present for every registered patient in a database established for rehabilitation research in secondary healthcare [20]. Degree of data completeness for longitudinal data has been assessed, mostly in terms of methods for handling missing data occurring completely at random, at random, or not at random [21–23]. However, the completeness of digital data collected in rehabilitation research settings has seldom been explored. In addition, there is a need for knowledge about factors related to missing PROM data, such as the length of data sets, patterns of response, and characteristics of patients, rehabilitation institutions, and data collection routines [18, 24]. Such knowledge may be valuable in efforts to ensure data completeness and support stakeholders’ processes of selecting appropriate sets of PROMs for use in patient records, clinical trials, or rehabilitation registries.

Therefore, the overall aim of this study was to analyse response patterns and the completeness of a brief set of PROMs compared with a longer set of PROMs. PROM data were generated digitally as repeated measurements along the course of rehabilitation from secondary healthcare throughout the subsequent follow-up period at home. We hypothesized that the degree of data completeness in systematic outcome assessments in rehabilitation research will improve when the number of questionnaires is kept to a minimum.

## Methods

### Study design and clinical setting

**Fig. 1:**
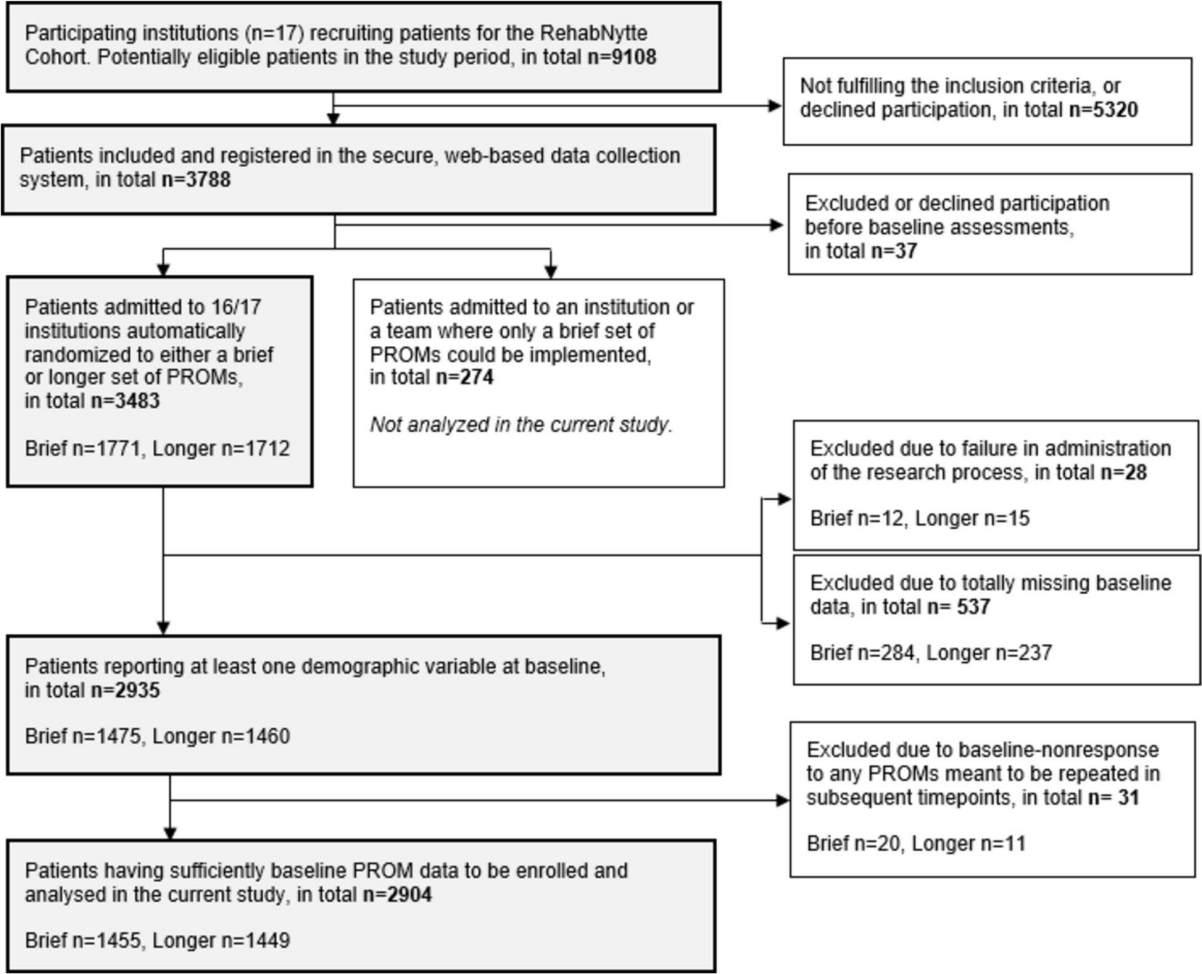
Flow chart of the progression from eligible patients to enrolment in the current study

This multicentre randomized controlled trial was initiated by several rehabilitation managers who were members of the Rehabilitation Research and Development Network in the VIRKE Enterprise Federation in Norway. Members of the Network wanted to explore the relationship between data completeness and length of provided questionnaire packages. The Network also sought to establish a secure digital database, the RehabNytte Cohort, for monitoring patient engagement, the quality of rehabilitation services, patients’ progress on PROMs, and their overall benefits of rehabilitation services. These objectives were integrated into the design of the RehabNytte Project [25]. Development of the longitudinal multicentre RehabNytte Cohort is presented in Table 1. The RehabNytte Project was designed to enable a combination of a randomized controlled trial (the current study) and a cohort approach (several other research questions) for the purpose of multiple research questions. Accordingly, when included in the RehabNytte Cohort, patients were randomized to complete either a brief or a longer set of PROMs at five different time points during a year (Fig. 1).

**Table 1:** The longitudinal multicentre RehabNytte Cohort

**The RehabNytte (RehabBenefit) Cohort** included adults admitted within the project period to one of 17 private rehabilitation centres contracted by Norway’s public health authorities and part of the VIRKE Rehabilitation Research and Development Network. All centres provided multidisciplinary rehabilitation services within Norway’s secondary healthcare system	
Overall aim	To monitor patient engagement, rehabilitation quality, and patients’ progress and benefit of rehabilitation services on work ability, health, functioning, and well-being
The three work packages	Patient engagement in rehabilitation (1). Rehabilitation and work participation (2). Quality of services and patient-reported outcomes rehabilitation (3). The current study is on of several studies belonging to work package 3
Inclusion period	Patients were included in the period from January 2019 to March 2020, and followed for 12 months. Data collection was completed in June 2021
Inclusion criteria	Patients were eligible for inclusion across diagnosis, but needed to be ≥ 18 years of age, and referred by a general practitioner or a specialist physician to multidisciplinary rehabilitation in secondary healthcare at one of the participating centres. Due to digital and self-reported data collection, they had to be able to read and understand questionnaires in Norwegian, have access to a personal computer, tablet, or smartphone, and have a personal electronic credential for secure identification online Patients with severe cognitive impairment(s) or psychiatric disease(s) influencing their ability to perform self-reported assessments over time were excluded
Data collection	Patient-reported outcome measures were collected digitally at admission (T1), discharge (T2), and 3 (T3), 6 (T4), and 12 (T5) months post-admission, through a General Data Protection Regulation-compliant web system with level 4 data-security Health professionals recorded referral diagnosis and registered patients using their national identification number
Interventions	The centres had different target groups and facilities, but all delivered rehabilitation programs involving at least four professionals, including nurses, physiotherapists, occupational therapists, and medical doctors, with some teams also including social workers, dietitians, sport educators, or psychologists. Most centres provided inpatient stays for 2–4 weeks, while one centre offered a 1-week outpatient program, and another offered a return-to-work program lasting up to six months. Sessions, both individual and group-based, focused on patient education, disease information, coping strategies (e.g., for fatigue, pain, sleep, stress), and healthy lifestyle changes (e.g., physical activity and training, activities of daily living, weight management, smoking cessation). Additional support covered family and social relationships, work adaptations, and social services. The programs were tailored to meet each patient’s needs and goals, collaboratively agreed on in meetings between the patient and members of the team
Outcomes	Measured at T1: Sociodemographic variables Measured at T1-T5: Self-assessed work ability, pain, functioning, health-related quality of life, rehabilitation goal(s) and goal attainment(s) Measured at T2-T5: Self-assessed change in health status (benefit of rehabilitation) and the acceptability of present health status Measured at T3 and analysed in another study: Patient experience feedback on quality of the received rehabilitation service
Design and participating centres in the current study	The current randomized controlled trial was planned as an independent study within the RehabNytte Project, aiming to examine the relationship between data completeness and length of provided questionnaire-set. Patients were included at 16 out of 17 RehabNytte centres

Participating centres were private rehabilitation institutions contracted to the public health authorities and part of the secondary healthcare system in Norway. Patients included in the RehabNytte Cohort were in need of multidisciplinary rehabilitation in secondary healthcare due to various chronic diseases, such as rheumatic or musculoskeletal disease, cardiovascular disease, neurological disease, or cancer. Patients with other chronic diseases were also eligible, as the study aimed to reflect the disease heterogeneity characterizing the adult population in need of rehabilitation in secondary healthcare. Patients referred to secondary healthcare generally need more intensive rehabilitation compared to those receiving rehabilitation in primary care. This often involves broad access to multidisciplinary health professionals with specialized education and expertise [26, 27].

Included patients in the RehabNytte Project were admitted to one of 17 participating institutions in the period from January 2019 to March 2020. They completed PROM data five times over a one-year period. The final follow-up was completed in June 2021. At one institution, only a brief set of PROMs could be implemented. As a result, this randomized controlled trial was conducted at 16 out of 17 RehabNytte institutions, where it was feasible to randomize patients to complete either a brief or a longer set of PROMs.

Written, informed consent to participate was based on verbal and written information about the study. Patient research partners and clinician-representatives were involved in all stages of the study. The study was considered by the Norwegian Regional Committee for Medical Research Ethics as not requiring approval, because of its overarching goal to evaluate the delivery of rehabilitation services (2018/1645/REK South-East A). It was recommended by the data protection officer at Diakonhjemmet Hospital (DS-00040), dated 17.10.2018, and registered in ClinicalTrials.gov (NCT03764982) [25].

### Data collection and measurements

Data were digitally collected at admission (T1), discharge (T2), and after 3 (T3), 6 (T4), and 12 (T5) months through a system approved in accordance with EU General Data Protection Regulation. At each time point except T2, patients received an automated email notification and a text message on their mobile phones including the link to the electronic data collection system. Non-responders received an automated reminder via email and text message after one week. No automated notifications or reminders were used at T2, due to various length of stay within and across the rehabilitation institutions, but clinicians at participating centres were requested to ask their patients to log in and complete the T2 assessments while they were still at the institution.

At T1, patients in both groups first responded to 14 sociodemographic variables. Further, throughout the study period (T1–T5), the patients responded to repeated measurements addressing the patient perspectives on work ability, pain, and health-related quality of life. In the period from T2 to T5, two additional questions comprised self-assessed change in health status (benefit of rehabilitation) and the acceptability of present health status. Patients randomized to complete the longer set of PROMs additionally responded to questions about patient-specific rehabilitation goals, goal attainment, and function.

To assess *work ability*, we used the Work Ability Score from the Work Ability Index [28, 29]. Patients responded to the single item “current work ability compared with the life-time best” on an 11-point numeric rating scale from 0 = “unable to work” to 10 = “work ability as its best” [30].

*Pain* was assessed using the single question “Do you have pain?” (dichotomous, yes/no). If yes, additional items were (1) “Duration of pain for 3 months or longer?” (dichotomous, yes/no); (2) “Distribution of pain” (dichotomous, pain located to one area of the body *versus* several areas of the body); and (3) “How would you describe your pain in the last week?” (11-point numeric rating scale from 0 = “no pain” to 10 = “worst possible pain”) [31, 32].

Patient-reported rehabilitation benefit and progress over time were assessed in terms of perceived *change in health status* using the Global Rating of Change Scale (GRC) [33]. Patients responded on a 5-point scale to a single question about whether their perceived health status was changed compared with the pre-rehabilitation status (“Compared to before rehabilitation, do you feel that your current health is…much worse/worse/unchanged/improved/much improved”) [33].

*Acceptability* was assessed by the Patient Acceptable Symptom Scale [34, 35]. Patients responded to a dichotomized single-item question as to whether their present health status (in the last week) could be considered acceptable if it were to continue without change (in the next months) (response options: acceptable/unacceptable) [35, 36].

The multi-item EQ-5D-5L questionnaire, developed by the EuroQol Group, was used to assess *health-related quality of life* (HRQoL) [37]. Patients were asked to describe their health that day on five dimensions: mobility, self-care, usual activities, pain/discomfort, and anxiety/depression [38]. For each dimension, patients responded to five possible levels (response categories): no problems, slight problems, some/moderate problems, severe problems, unable to perform/extreme problems [38]. Patients also rated their health state on the EQ VAS, a visual analogue scale from 0 indicating “the worst health you can imagine” to 100 indicating “the best health you can imagine” [38].

In addition to the instruments mentioned above, the following two instruments were included in the longer set of PROMs:

The Patient-Specific Functional Scale (PSFS) [39] was used to identify *patient-specific rehabilitation goal(s)* and assess potential *goal attainment(s).* At T1, patients were asked to identify up to five activities they found difficult to perform due to their health condition [40, 41]. For each activity, they were asked to rate current performance on a numeric rating scale from 0 indicating “unable to perform activity” to 10 indicating “able to perform activity at the same level as before injury or disease” [40, 41]. They thereafter rated performance of the same activities at T2–T5.

To assess broader aspects of *functioning*, we used the 29-item version of the PROMIS Profile instruments (PROMIS-29) developed by the National Institutes of Health [42–44]. Patients reported on their present ability regarding two health domains (physical function, and satisfaction with participation in social roles and activities), and experiences over the previous 7 days regarding five domains (anxiety, depression, fatigue, sleep disturbance, and pain inferences) [42]. Each domain comprises 4 items (in total, 28 items), each using a 5-category response scale labelled to be relevant for each domain: for example, not at all/a little bit/somewhat/quite a bit/very much [42]. Lastly, PROMIS-29 include an 11-point numeric rating scale assessing pain intensity from 0 = “no pain” to 10 = “worst pain imaginable” [42].

As some data items were applicable under only certain conditions, the numbers of items expected to have values were stated as ranges. Thus, the total number of questions to be answered by patients receiving the brief set was 8–11 at T1, and 10–13 for each time point from T2 to T5. For patients receiving the longer set, the number of questions to be answered was 38–45 at T1, and 40–47 for each time point T2–T5. The information letter provided to the patients stated that each assessment event was estimated to take between 5 and 20 min to complete.

### Randomization

The recruitment period for the RehabNytte project was set from January 2019 to March 2020, with an estimated 9000 eligible patients. Assuming that approximately 50% of invited patients agreed to participate, we anticipated enrolling 4000 to 4500 participants, a number considered adequate to detect potential differences between the two groups in degree of completeness and response patterns.

The randomization into either brief or longer set of PROMs was conducted through a computerized process, utilizing a randomization service integrated into the online data collection system. The automatic group allocation was determined by the date on which a local health professional registered a patient in the online database, prior to the patient’s admission to the institution. This algorithm permitted randomization at any inclusion time during the study enrolment period, without involvement of any persons who included, assessed, treated, or evaluated the patients or their research data. The system ensured that neither the health professionals nor the patients could predict the group allocation ahead of assignment. A minimization or stratification method was not used. Although the allocation to a brief or longer set of PROMs was not stated in the information letter, the patients were aware that they were participating in a clinical trial and were informed about the study’s objectives, including the use of various PROMs.

### Statistical analysis

For the purpose of this study, we aimed to use PROM data collected at T1 as a benchmark to evaluate subsequent repeated PROMs captured at T2-T5. Therefore, prior to any analyses, we excluded data entries that lacked any corresponding PROM data at T1 [45].

We measured completeness as percentages, in terms of the present data as a proportion of the potential 100% completeness expected to be reported for applicable variables in the database [20]. However, during the analyses, we considered the conversion from continuous data (percentage) to categorical data (fully completed, partly completed, fully missing) as most appropriate. This was decided after initial analyses disclosing a high proportion of accumulated responses as either complete responses or completely missing. Thus, results from the initial analysis violated the assumption of continuously or normally distributed data on the degree of completeness.

The hypothesis concerning completeness was tested at the group level, focusing on the overall completeness for the data sets. At each timepoint (T1-T5), we compared the brief and longer data set groups in terms of the proportions of: (1) fully completed data sets (all applicable items answered), (2) partly completed data sets (at least one but not all applicable items answered), and (3) completely missing responses (no items answered).

Potential differences in response patterns were analysed at the individual level, examining each participant’s longitudinal response across the study period (T1-T5). We compared the brief and longer data set groups based on the proportions of: (1) participants completing or partially completing responses at all time points (T1-T5), and (2) participants having a response dropout for the remainder of the study period, categorized as: lost at discharge (missing data T2-T5), lost at 3 months (missing data T3-T5), lost at 6 months (missing data T4-T5), and lost at 12 months (missing data T5)). The third response pattern was (3) participants who missed one or more assessment event(s) but later resumed responding. These intermittent missing assessments were classified into the following categories: missing only at discharge, missing only at 3 months, missing only at 6 months, missing at two time points within the period T2-T5, and missing at three time points within T2-T5.

Finally, we conducted multivariate logistic regression analyses to examine whether patients’ characteristics, admission centre, referral diagnosis, symptoms, and/or functional levels influenced the degree of completeness. Specifically, we analysed the probability of giving a response (complete or partial) at the last time point (T5) based on multiple sets of variables. The first set of variables included patients’ sociodemographic characteristics. Next, we added a second set of variables capturing their work ability, pain, functional status, and HRQoL. Third, we included a set of variables related to the allocation of patients across the various participating rehabilitation centres. This analysis was conducted at time point T2, in addition to T5, because initial analyses disclosed important differences in completeness between institutions at T2. In all regression analyses, we used a binary outcome to determine whether a patient had responded at T5 (either all items or at least one) or not (no items answered). The independent variables were also converted into binary outcomes to facilitate the examination of odds ratios. We used a binary depended outcome because the initial analyses revealed that the proportion of partially completed responses was low, leading us to combine complete and partially completed responses into the same category.

We used STATA IC, version 17.0, for statistical analyses, and set the statistical significance level to 0.05.

## Results

A total of 2904 patients were included and randomly assigned to complete either the brief (1455 patients, 50.1%) or the longer (1449 patients, 49.9%) set of PROMs. Figure 1 illustrates the progression from identifying eligible patients to their final enrolment in the current study.

For the total sample, the mean age of patients was 53.6 years (standard deviation (SD): 13.8), 69.4% were female, 46.8% had higher education, 93.5% had a Scandinavian country of origin, and 56.2% were currently in paid work (full time or part time). The patients were referred to rehabilitation most frequently due to rheumatic and musculoskeletal diseases (39.3%) or cancer (26.9%) (Table 2). In total, 78.0% of the patients had pain, mostly spread to several areas of the body (66.9%), with duration ≥ 3 months (90.4%). The between-group comparability was acceptable for all sociodemographic baseline variables (Table 2). The degree of data completeness for demographic variables was high (ranging from 91.0 to 99.9%) and comparable between groups (Table 3).

**Table 2:** Baseline characteristics of patients (*n* = 2904) and their allocation across rehabilitation institutions (*n* = 16)

	**Group 1 Brief set of PROMs **(*n* = 1455)	**Group 2 Longer set of PROMs **(*n* = 1449)
Age^a^, years, mean (SD)	53.2 (13.6)	54.0 (13.9)
Sex^a^, female, %	1006 (69.1)	1008 (69.6)
Referral diagnosis^b^, *n* (%)		
Rheumatic or musculoskeletal diseases	586 (40.3)	556 (38.4)
Cancer	372 (25.6)	408 (28.2)
Neurological disease	170 (11.7)	179 (12.4)
Lifestyle disease, overweight	148 (10.2)	125 (8.6)
Sensory impairment	55 (3.8)	66 (4.6)
Cardiovascular disease	44 (3.0)	51 (3.5)
Mental disease	16 (1.1)	10 (0.7)
Other disease	64 (4.4)	54 (3.7)
Institution^b^, *n* (%)		
Centre 1	57 (3.9)	63 (4.4)
Centre 2	133 (9.1)	147 (10.1)
Centre 3	34 (2.3)	35 (2.4)
Centre 4	95 (6.5)	76 (5.2)
Centre 5	71 (4.9)	48 (3.3)
Centre 6	191 (13.1)	199 (13.7)
Centre 7	100 (6.9)	116 (8.0)
Centre 8	23 (1.6)	16 (1.1)
Centre 9	21 (1.4)	20 (1.4)
Centre 10	94 (6.5)	83 (5.7)
Centre 11	26 (1.8)	21 (1.5)
Centre 12	30 (2.1)	28 (1.9)
Centre 13	65 (4.5)	57 (3.9)
Centre 14	155 (10.7)	139 (9.6)
Centre 15	57 (3.9)	59 (4.1)
Centre 16	303 (20.8)	342 (23.6)
Patient-reported data		
Comorbidities, *n*, median (min, max)	2 (0,10)	2 (0,10)
Body mass index, kg/m^2^, mean (SD)	29.5 (7.0)	29.1 (6.7)
Smoking and/or snuff use, *n* (%)	322 (22.1)	342 (23.6)
Education > 12 years, *n* (%)	682 (46.9)	679 (46.9)
Paid work (currently, full or part time), *n* (%)	839 (57.7)	794 (54.8)
Recipients of social security benefits, *n* (%)	1173 (80.6)	1187 (81.9)
Language (native tongue), *n* (%)		
Norwegian, Swedish, or Danish	1363 (93.7)	1378 (95.1)
Other languages	89 (6.1)	67 (4.6)
Country of origin, *n* (%)		
Norway, Sweden, or Denmark	1350 (92.8)	1364 (94.1)
Other countries	100 (6.9)	82 (5.7)
Civil status, *n* (%)		
Married / cohabitant	925 (63.6)	902 (62.3)
Single	528 (36.3)	544 (37.5)
Caregiver for child(ren)/others in or outside home, *n* (%)	648 (44.5)	623 (43.0)
Annual gross income in the household > 600 000 NKr, *n* (%)	708 (48.7)	680 (46.9)

*SD* Standard deviation

^a^Data collected from the national identification number

^b^Clinician-reported data, mandatory

**Table 3:** Degree of completeness of patient-reported demographic baseline data

Complete response = yes, *n* (%)	**Group 1 Brief set of PROMs **(*n* = 1455)	**Group 2 Longer set of PROMs **(*n* = 1449)	**Difference between groups**^**a**^(*p*-value)
Comorbidities *(multiple choice category, 19 choices)*	1324 (91.0)	1333 (92.0)	0.34
Body mass index *(open categories, body weight and height)*	1442 (99.1)	1424 (98.3)	0.05
Smoking and/or snuff use* (multiple choice category, 4 choices)*	1455 (100.0)	1448 (99.9)	0.32
Education *(multiple choice category, 3 choices)*	1453 (99.9)	1446 (99.8)	0.65
Status of paid work & social benefits *(multiple choice category, 9 choices)*	1453 (99.9)	1147 (99.9)	0.99
Language *(open category)*	1452 (99.8)	1445(99.7)	0.70
Country of origin *(open category)*	1450 (99.7)	1446 (99.8)	0.48
Civil status *(dicohotomous category)*	1453 (99.9)	1446 (99.8)	0.65
Caregiver for child(ren)/others in or outside the home *(dicohotomous category)*	1454 (99.9)	1448 (99.9)	0.99
Annual gross income in the household *(multiple choice categories, 4 choices)*	1443 (99.2)	1432 (98.8)	0.35

^a^Pearson chi-square test

### Data set completeness

**Fig. 2:**
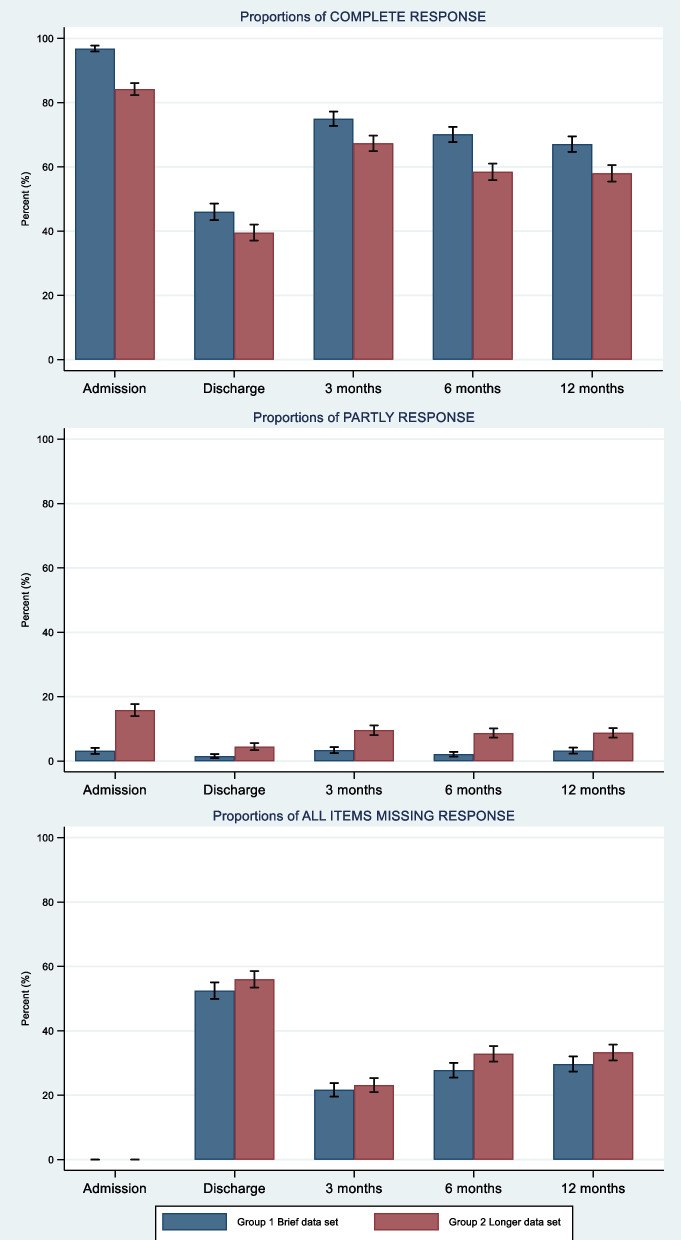
Proportions of complete, partly and missing response for the entire data set (group level), with 95% confidence intervals

At each time point, data set completeness was statistically significantly higher for Group 1 (brief set) compared to Group 2 (longer set) (*p* < 0.001; Fig. 2, upper panel). At admission, the estimated completeness in the brief set was 96.8% (95% confidence interval (CI) 0.96–0.98) compared to 84.2% (95% CI 0.82–0.86) in the longer version. At discharge the numbers were 46.0% (95% CI 0.43–0.49) in the brief set compared to 39.5% (95% CI 0.37–0.42) in the longer. In the follow-up, the completeness was 75.0% (95% CI 0.73–0.77) (brief) compared to 67.3.0% (95% CI 0.65–0.70) (longer) at T3, 70.1% (95% CI 0.68–0.72) (brief) compared to 58.5.0% (95% CI 0.56–0.61) (longer) at T4, and 67.1% (95% CI 0.65–0.69) (brief) compared to 58.0% (95% CI 0.55–0.60) (longer) at T5. Across the study period, the observed differences between groups ranged from 6.5 percentage points (at discharge) to 12.6 percentage points (at admission), consistently favouring the brief set (Fig. 2, upper panel).

The overall pattern of completeness was similar between the groups, with the highest level of data set completeness observed at admission and the lowest at discharge. Within the follow-up period, the level of completeness decreased slightly in both groups, but the change from T3 (3 months) to T5 (12 months) was < 10 percentage points within each group (Fig. 2, upper panel). The frequency of partially completed measurement events was higher in the longer data set compared to the brief version. However, such instances remained relatively rare in both groups when compared to the proportion of fully completed or entirely missing responses (Fig. 2).

### Response patterns

**Fig. 3:**
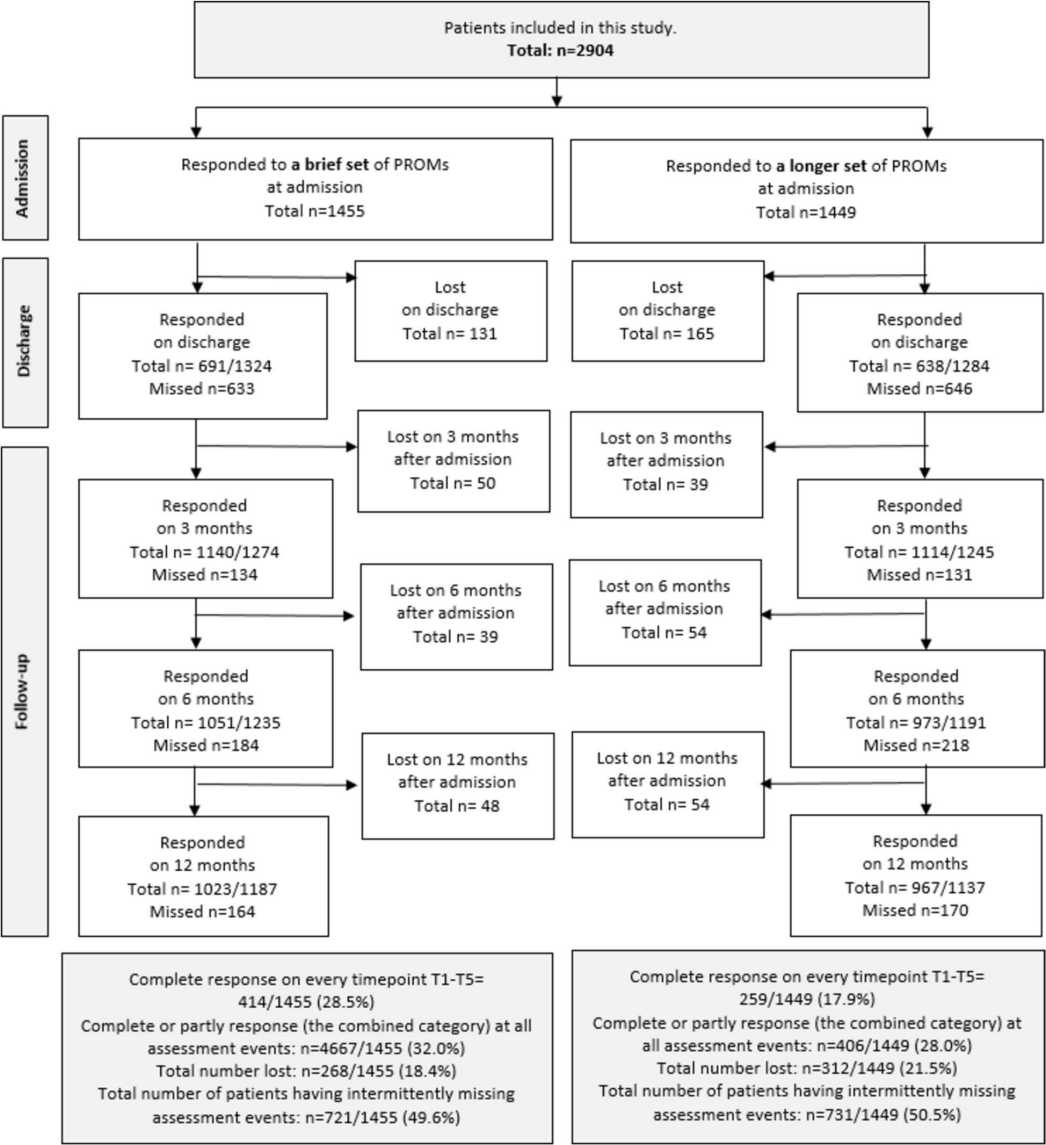
Flow diagram of participants, summing up information regarding the observed response patterns

At the individual level, the proportion of patients with fully complete data set responses at every time point was low in both groups. However, it was statistically significantly higher in Group 1 (brief data set) at 28.5%, compared to 17.9% in Group 2 (longer version) (*p* < 0.001) (Fig. 3). The difference between groups was smaller but remained statistically significant for the proportion of patients with any response (a combined category of partial or complete responses) at all time points, with 32.0% in the brief set compared to 28.0% in the longer version (*p* = 0.02).

Across all time points (T1-T5), the dropout rate was statistically significantly lower in Group 1 (brief) at 18.4% compared to 21.5% in Group 2 (longer) (21.5%) (*p* = 0.04).

The degree of intermittent missing data was high and comparable between groups, at 49.6% and 50.5%, respectively. The highest proportion of intermittent missing data was observed in the category “Missing assessment on discharge only”, being 26.4% of the brief data set group and 25.1% of the longer version group.

In Group 1 (brief) 25 out of the 268 dropouts (9.3%) were explicit withdrawals, with 8 individuals providing a stated reason. In Group 2 (longer version) 28 out of the 312 dropouts (9.0%) were explicit withdrawals, with 13 individuals providing a stated reason. The reasons were comparable between groups and included “other illness”, “interrupted rehabilitation stay before discharge”, “technical login problems “, “lack of computer skills”, “lack of motivation”, “assessments perceived as too comprehensive”, and “language impairment”. Reasons for intermittently missed assessment events were not reported.

### Instrument completeness

Instrument completeness was comparable between groups for EQ-5D-5L and other instruments included in both data sets (Appendix 1, Fig. 1). Completeness for the additional instruments in the longer data set was comparable, but slightly lower: The PROMIS-29-completeness at T1 was 4.8 percentage lower than for EQ-5D-5L (98%). During follow-up, PROMIS-29-completeness was 3.3–3.7 percentage points lower than for EQ-5D-5L, which ranged from 75% at T3 to 65% at T5. For PSFS compared to EQ-5D-5L, the completeness was 7.5 percentage points lower at T1 and 1.3–2.4 percentage points lower during T3-T5 (Appendix 1, Fig. 2). Among all instruments, the degree of non-response during the study period was highest for PSFS. The degree of partial response was highest for PROMIS-29 (Appendix 1, Table 1).

Among participants in Group 2 (the longer data set) who completed every EQ-5D-5L item, over 90% also responded to all the PROMIS-29-items: 93.7% at T1, 96.0% at T2, 94.2% at T3, 92.9% at T4, and 93.1% at T5.

### Regression analysis

In the logistic regression analyses focusing on the 12 months follow-up, there were positive associations between being a responder and higher age (odds ratio (OR) 1.02, 95% CI 1.01–1.03), female gender (OR 1.39, 95% CI 1.17–1.67), higher education (OR 1.21, 95% CI 1.02–1.45), and being Scandinavian (OR 2.11, 95% CI 1.15–3.89). Conversely, negative associations were observed with smoking or snuff use (OR 0.65, 95% CI 0.53–0.79), being single (OR 0.80, 95% CI 0.65–0.99), and being a caregiver for children or others either in or outside the home (OR 0.81, 95% CI 0.68–0.97). Although the OR for the age variable was close to 1, we included it along with the other six sociodemographic variables in the subsequent analysis. When we added variables related to the referral diagnosis, patient-reported work ability, pain, functional status and HRQoL, we observed a negative association between being a responder and having moderate to extreme problems with anxiety or depression, as measured by EQ-5D-5L. Finally, when adding variables capturing the allocated rehabilitation centre, negative associations were observed with allocation to two out of 16 centres. All regression analysis results are presented in Appendix 2, Table 1–3.

Since no automated reminders were sent to non-responders at discharge, this time point could offer additional insights into how the centres may serve as contextual factors influencing the participants’ response rates. We observed that the data completeness across participating centres at discharge (T2) ranged from 4.9% to 81.0% (difference (∆) of 76.1 percentage points), compared to a range between 83.2% and 95.3% (∆ = 11.5) at admission (T1). In the follow-up period, the completeness ranged between 55.3% and 82.9% at T3 (∆ = 27.6), between 46.8% and 71.0% at T4 (∆ = 24.2), and between 51.3% and 74.1% (∆ = 22.8) at T5.

## Discussion

This study demonstrated that a brief set of PROMs achieved higher data completeness than a longer set, when used for repeated measurements in adults over a one-year follow-up in a rehabilitation research setting.

At rehabilitation admission, the data set completeness was high (≥ 84.2%) in both groups. This level of baseline completeness was comparable to response rates in Scandinavian registries (84.2–96.8% versus 73 ± 25.4%, respectively) [46]. Also consistent with previous findings [46], completeness during the follow-up assessments (T3–T5) declined or plateaued in both groups. Contrary to our expectations, though, the lowest degree of completeness was observed at discharge (39.5–46.0%). This may be attributed the absence of automatic reminders at discharge, and the possibility that clinicians or administrative staff forgot to remind patients to complete PROMs while they were still at the institution. These findings align with previous research showing that reminders are critical for improving patients’ response rates in repeated assessments over extended periods [46]. Our results support the notion that failure in logistic factors results may contribute more significantly to missing PROM data than patient-related issues [7]. Therefore, strategies to prevent or minimize missing data must extend beyond reducing patient burden. Future attention should address robust administrative leadership to ensure optimal adherence to data collection routines [18, 45, 46].

In our study, the proportion of patients with fully complete responses at all five scheduled time points was 10.6% higher for the brief set compared to the longer set. The lower completeness observed for the longer data set was partly due to a higher dropout rate (3.1 percentage points higher than the brief set) and partly due to a larger number of missing items within single assessment points (6.6 percentage points higher). Researchers should determine an acceptable level of missing data based on the purpose of their study or registry. The literature offers various statistical methods for handling missing PROM data to mitigate biased conclusions and ensure validity [47, 48]. However, these methods may not be suitable for all type of instruments, for instance PSFS or other patient-specific instruments with open-ended categories. In our study, the degree of non-response was highest for PSFS. Some patients may have encountered difficulties in scoring their performance on the self-selected activities. Other cases of non-response could stem from baseline-selected activities becoming less relevant or inapplicable as rehabilitation goals during the study period [41, 49]. While additional questions or instruments in a set of outcomes may be relevant for researchers or other stakeholders, the benefit of a broader data collection is limited if the dropout rate becomes higher. As pointed out in a scoping review, strategies to ensure complete PROM data are even more important in studies with several assessment events over an extended period of time, in comparison with single time point surveys [46].

The PROMIS-29 had the highest degree of missing items compared to other instruments in our study. Based on existing evidence, the degree of completeness may be negatively influenced by overlapping health domains [13]. The overlapping domains between EQ-5D-5L and PROMIS-29 may have resulted in a higher patient burden for those assigned to the longer data set. In the future, the availability of a Norwegian item bank could enable the administration of the PROMIS-29 as a computerized adaptive test. This may reduce the length of the data set and minimize the risk of overlapping or irrelevant content by presenting only the most informative questions based on the patient’s previous responses [50]. On the other hand, the opportunity to elaborate on important health issues may be appreciated and meaningful for patients, as long as questions addressing the same topic are complementary and not identical [45]. This may explain why over 90% of those responding to all EQ-5D-5L-items also completed all items in PROMIS-29. Still, reducing the patient burden is emphasized in systematic reviews as an important strategy to prevent dropouts and ensure complete PROM data [18, 45].

### Strengths and limitations

Strengths of this study include the randomized set-up in real-life clinical practice, which reflects the disease heterogeneity among patients in need of rehabilitation. Also, the use of generic PROMs strengthens the study, as all the included instruments are applicable and commonly used in rehabilitation research and registries across patient groups and nations. The study could have been strengthened by defining an expected difference in completeness when adding PSFS and PROMIS-29 to the brief data set. However, our aim was to explore differences rather than to target a specific magnitude of difference. A limitation is that the study may be underpowered, as the total number of participants included in the randomized controlled trial was lower than the expected number of 4000. Moreover, blinding participants to their group allocation was not feasible, as they could have observed that other patients were completing a different set of questionnaires. Similarly, it was not possible to blind health professionals, especially if patients in the group with the longer questionnaires sought assistance with questionnaires specific to that group (e.g., PSFS or PROMIS-29). Furthermore, the generalizability of the results may be limited to study settings, as the use of PROMs in routine clinical practice could differ from their use in research.

Finally, our study was restricted to data completeness, which is only one aspect of data quality [20]. Other dimensions, such as accuracy and consistency of data values for the relevant PROMS, should also be considered when planning data collection for clinical trials or registries. A persistent and highlighted attention to data quality is called for in future, including efforts to prevent avoidable missing values, and strategies to ensure optimal administration routines [45].

## Conclusion

This randomized controlled trial on PROM data completeness identified a statistically significant improvement in data completeness for a brief PROM dataset compared to a longer one. To optimize the use of PROMs in clinical trials and registries, stakeholders should carefully balance the necessary content and length of data sets to align with their intended purpose and relevance. Strategies to minimize the degree of missing data or dropouts should include the use of reminders for non-responders, strong administrative leadership, and optimal data collection routines.

## Supplementary Information

Supplementary Material 1.

Supplementary Material 2.

## Data Availability

Data is provided within the manuscript or appendixs. The dataset utilized and analysed can be obtained from the corresponding author upon a reasonable request, restricted to parts with permission from the Norwegian Regional Committee for Medical Research Ethics (REK South-East).

## Abbreviations

PROMs: Patient-reported outcome measures
EQ-5D-5L: The multi-item EQ-5D-5L questionnaire, developed by the EuroQol Group
HRQoL: Health-related quality of life
PSFS: The Patient-Specific Functional Scale
PROMIS-29: The 29-item version of the PROMIS Profile instruments developed by the National Institutes of Health
SD: Standard deviation
T1: At admission
T2: At discharge
T3: 3 Months after admission
T4: 6 Months after admission
T5: 12 Months after admission
