# Supplementary material for: Completeness of repeated patient-reported outcome measures in adult rehabilitation: a randomized controlled trial in a diverse clinical population
Source: BMC Health Serv Res. 2024 Dec 24;24:1648. doi: 10.1186/s12913-024-12103-8 (PMC11668074; doi:10.1186/s12913-024-12103-8)
Supplement: Supplementary file 1 — Supplementary Material 1. [file 12913_2024_12103_MOESM1_ESM.docx]

**Appendix 1 Instrument Completeness**


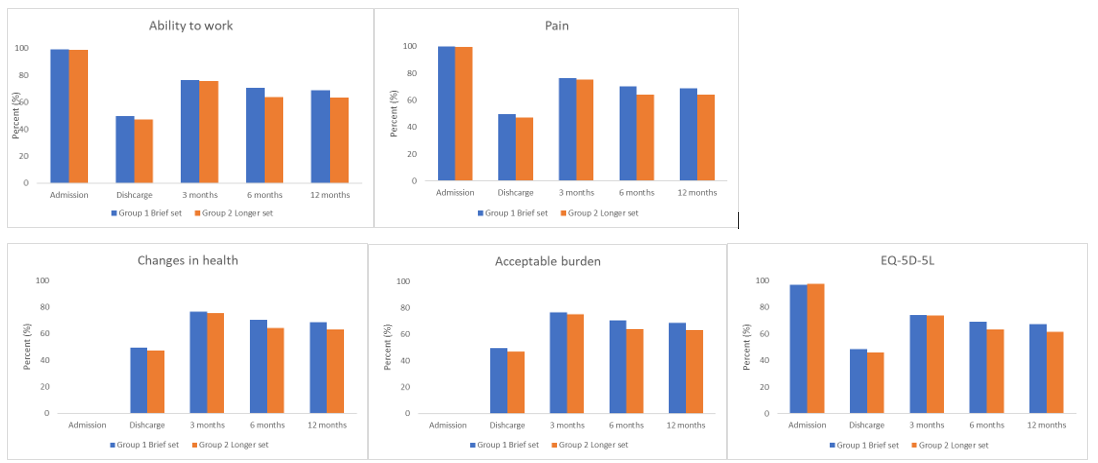


**Figure 1** Instrument completeness (best=100%) in the brief set of patient-reported outcome measures compared to the same measures in the longer set.

**Figure 2** The proportions of patients with totally complete responses to the specific instruments included in the study. *PROMs = patient-reported outcome measures. To improve readability, data labels for EQ-5D-5L bars are shown.*

**Table 1** Proportion (%) of levels of instrument completeness during the course of rehabilitation and follow-up.

| Questionnaire | Response | **Group 1 Brief data set** | | | | | **Group 2 Longer data set** | | | | |
| --- | --- | --- | --- | --- | --- | --- | --- | --- | --- | --- | --- |
|  |  | T1 | T2 | T3 | T4 | T5 | T1 | T2 | T3 | T4 | T5 |
| **Ability to work**  (single item) | Complete response | 99,5 | 47,3 | 78,1 | 72,1 | 69,8 | 99,2 | 43,7 | 76,5 | 66,7 | 66,5 |
|  | Non-response | 0,6 | 52,7 | 21,9 | 27,9 | 30,2 | 0,8 | 56,3 | 23,5 | 33,3 | 33,5 |
| **Changes in health**  (single item) | Complete response | n.a | 47,2 | 78,0 | 72,0 | 69,8 | n.a | 43,6 | 76,5 | 66,9 | 66,3 |
|  | Non-response | n.a | 52,8 | 22 | 28 | 30,2 | n.a | 56,4 | 23,5 | 33,1 | 33,8 |
| **Acceptable burden**  (single item) | Complete response | n.a | 47,1 | 77,9 | 71,8 | 69,5 | n.a | 43,6 | 76,3 | 66,7 | 66,2 |
|  | Non-response | n.a | 52,9 | 22,1 | 28,2 | 30,5 | n.a | 56,5 | 23,7 | 33,3 | 33,8 |
| **Pain**  (multi items) | Complete response | 99,8 | 47,3 | 78 | 71,9 | 69,9 | 99,7 | 43,6 | 76,4 | 66,7 | 66,2 |
|  | Partly response | 0,1 | 0 | 0,1 | 0,2 | 0,1 | 0,1 | 0,2 | 0,1 | 0,1 | 0,1 |
|  | Non-response | 0,1 | 52,7 | 21,9 | 27,9 | 30 | 0,1 | 56,3 | 23,5 | 33,2 | 33,7 |
| **EQ-5D-5L**  (multi items) | Complete response | 97,5 | 46,3 | 75,7 | 70,4 | 68 | 97,8 | 42,9 | 74,7 | 65,7 | 64,5 |
|  | Partly response | 2,5 | 0,8 | 1,7 | 1,5 | 1,8 | 2,2 | 0,8 | 1,7 | 1,2 | 1,4 |
|  | Non-response | 0 | 53 | 22,6 | 28,1 | 30,2 | 0 | 56,3 | 23,7 | 33,1 | 34,1 |
| **PSFS**  (multi items) | Complete response | n.a | n.a | n.a | n.a | n.a | 90,3 | 41,9 | 72,3 | 63,5 | 63,2 |
|  | Partly response | n.a | n.a | n.a | n.a | n.a | 4,8 | 0,7 | 0,9 | 1 | 0,6 |
|  | Non-response | n.a | n.a | n.a | n.a | n.a | 5 | 57,4 | 26,8 | 35,5 | 36,2 |
| **PROMIS-29**  (multi items) | Complete response | n.a | n.a | n.a | n.a | n.a | 93,0 | 41,8 | 71,4 | 62,0 | 61,0 |
|  | Partly response | n.a | n.a | n.a | n.a | n.a | 6,1 | 1,7 | 4,3 | 4,6 | 4,8 |
|  | Non-response | n.a | n.a | n.a | n.a | n.a | 0,9 | 56,6 | 24,3 | 33,4 | 34,2 |

*n.a=not applicable. T1: admission, T2: discharge, T3: 3 months, T4: 6 months, T5: 12 months. PSFS: Patient Specific Functional Scale.*
