# Supplementary material for: Completeness of repeated patient-reported outcome measures in adult rehabilitation: a randomized controlled trial in a diverse clinical population
Source: BMC Health Serv Res. 2024 Dec 24;24:1648. doi: 10.1186/s12913-024-12103-8 (PMC11668074; doi:10.1186/s12913-024-12103-8)
Supplement: Supplementary file 2 — Supplementary Material 2. [file 12913_2024_12103_MOESM2_ESM.docx]

**Appendix 2 Regression analysis**

**Table 1** Comparison of Responders (complete or partly) and Non-responders at the last time point (T5) in the follow-up period: Odds ratios from logistic regression analysis, part one.

| Variable | | Odds Ratio | 95% CI | p-value |
| --- | --- | --- | --- | --- |
| Age (per year increase) | | 1.02 | 1.01-1.03 | <0.001 |
| Female (vs male) | | 1.39 | 1.17-1.67 | <0.001 |
| Comorbidities (per number) | | 1.03 | 0.97-1.08 | 0.32 |
| Body Mass Index (per value, kg/m^2^) | | 1.00 | 0.99-1.02 | 0.62 |
| Smoking and/or snuff using (yes vs no) | | 0.65 | 0.53-0.79 | <0.001. |
| Higher education (> 12 years vs lower) | | 1.21 | 1.02-1.45 | 0.03 |
| Currently paid work (yes vs no) | | 1.14 | 0.95-1.38 | 0.16 |
| Recipients of social security benefits (yes vs no) | | 1.01 | 0.80-1.27 | 0.93 |
| Scandinavian language (yes vs no) | | 1.26 | 0.66-2.41 | 0.48 |
| Scandinavian country of origin (yes vs no) | | 2.11 | 1.15-3.89 | 0.02 |
| Civil status: Single (vs married or cohabitant) | | 0.80 | 0.65-0.99 | 0.04 |
| Caregiver for child(ren) / others in or outside home (yes vs no) | | 0.81 | 0.68-0.97 | 0.03 |
| Annual gross income in the household (>600 000 NKr vs lower) | | 1.10 | 0.89-1.37 | 0.36 |
| LR chi^2^ | 173.67 |  |  |  |
| Prob > chi^2^ | <0.001 |  |  |  |
| Pseudo R^2^ | 0.05 |  |  |  |
| *T5: follow-up at home 12 months after admission to rehabilitation centre, CI confidence interval* | | | | |

**Table 2** Comparison of Responders (complete or partly) and Non-responders at the last time point (T5) in the follow-up period: Odds ratios from logistic regression analysis, part two.

| **Variable** | | **Odds Ratio** | **95% CI** | **p-value** |
| --- | --- | --- | --- | --- |
| Age (per year increase) | | 1.02 | 1.01-1.03 | <0.001 |
| Female (vs male) | | 1.54 | 1.24-1.91 | <0.001 |
| Smoking and/or snuff using (yes vs no) | | 0.63 | 0.51-0.79 | <0.001 |
| Higher education (> 12 years vs lower) | | 1.23 | 1.01-1.50 | 0.04 |
| Scandinavian country of origin (yes vs no) | | 2.80 | 1.95-4.02 | <0.001 |
| Civil status: Single (vs married or cohabitant) | | 0.71 | 0.57-0.87 | <0.001 |
| Caregiver for child(ren) / others in or outside home (yes vs no) | | 0.80 | 0.66-0.99 | 0.04 |
| Widespread pain (yes vs no) | | 0.96 | 0.77-1.19 | 0.70 |
| Pain intensity (numeric rating scale score 5-10 (10=worst) vs score 0-1 (0=no pain) | | 1.12 | 0.87-1.43 | 0.37 |
| Ability to work (numeric rating scale score 5-10 (10=ability as its best) vs score 0-1 (0=not able to work) | | 0.97 | 0.76-1.23 | 0.78 |
| **EQ-5D-5L** | | | | |
| Mobility (moderate to extreme problems vs slight or no problems) | | 0.92 | 0.73-1.16 | 0.49 |
| Self-care (moderate to extreme problems vs slight or no problems) | | 1.16 | 0.82-1.64 | 0.40 |
| Usual activities (moderate to extreme problems vs slight or no problems) | | 0.98 | 0.78-1.23 | 0.87 |
| Pain / discomfort (moderate to extreme problems vs slight or no problems) | | 1.06 | 0.81-1.40 | 0.66 |
| Anxiety /depression (moderate to extreme problems vs slight or no problems) | | 0.78 | 0.62-0.96 | 0.03 |
| Overall self-rated health (visual analogue scale < 50 (0=worst health) versus ≥ 50 (100 best health) | | 1.18 | 0.95-1.47 | 0.13 |
| **Diagnosis** | | | | |
| Rheumatic or musculoskeletal diseases | |  |  |  |
| Cancer | | 1.03 | 0.61-1.73 | 0.92 |
| Neurological disease | | 0.79 | 0.46-1.35 | 0.39 |
| Lifestyle disease, overweight | | 1.30 | 0.74-2.29 | 0.37 |
| Sensory impairment | | 0.98 | 0.46-2.07 | 0.95 |
| Cardiovascular disease | | 1.36 | 0.60-3.06 | 0.46 |
| Mental disease | | 0.40 | 0.14-1.18 | 0.95 |
| LR chi^2^ | 181.81 | | | |
| Prob > chi^2^ | <0.001 | | | |
| Pseudo R^2^ | 0.066 | | | |
| *T5: follow-up at home 12 months after admission to rehabilitation centre, CI confidence interval* | | | | |

**Table 3** Comparison of Responders (complete or partly) and Non-responders at the last time point (T5) in the follow-up period: Odds ratios from logistic regression analysis, part three.

| **Variable** | | **Odds Ratio** | **95% CI** | **p-value** |
| --- | --- | --- | --- | --- |
| Age (per year increase) | | 1.02 | 1.01-1.03 | <0.001 |
| Female (vs male) | | 1.36 | 1.14-1.63 | <0.001 |
| Smoking and/or snuff using (yes vs no) | | 0.65 | 0.53-0.79 | <0.001 |
| Higher education (> 12 years vs lower) | | 1.25 | 1.06-1.49 | <0.001 |
| Scandinavian country of origin (yes vs no) | | 2.66 | 1.94-3.65 | <0.001 |
| Civil status: Single (vs married or cohabitant) | | 0.76 | 0.63-0.90 | <0.001 |
| Caregiver for child(ren) / others in or outside home (yes vs no) | | 0.81 | 0.68-0.98 | 0.03 |
| **Allocated rehabilitation centre** | | | | |
| Centre 1 (yes versus no) | | 0.90 | 0.57-1.40 | 0.64 |
| Centre 2 (yes versus no) | | 0.69 | 0.50-0.95 | 0.02 |
| Centre 3 (yes versus no) | | 1.10 | 0.62-1.93 | 0.75 |
| Centre 4 (yes versus no) | | 1.03 | 0.70-1.51 | 0.88 |
| Centre 5 (yes versus no) | | 1.24 | 0.79-1.94 | 0.36 |
| Centre 6 (yes versus no) | | 0.83 | 0.62-1.10 | 0.20 |
| Centre 7 (yes versus no) | | 0.86 | 0.60-1.23 | 0.41 |
| Centre 8 (yes versus no) | | 0.36 | 0.18-0.70 | <0.001 |
| Centre 9 (yes versus no) | | 0.56 | 0.29-1.08 | 0.08 |
| Centre 10 (yes versus no) | | 0.81 | 0.58-1.19 | 0.28 |
| Centre 11 (yes versus no) | | 1.06 | 0.55-2.05 | 0.86 |
| Centre 12 (yes versus no) | | 1.11 | 0.56-2.18 | 0.77 |
| Centre 13 (yes versus no) | | 1.08 | 0.69-1.69 | 0.73 |
| Centre 14 (yes versus no) | | 0.97 | 0.71-1.34 | 0.87 |
| Centre 15 (yes versus no) | | 1.44 | 0.89-2.34 | 0.14 |
| Centre 16 (yes versus no) | | 0.69 | 0.43-1.13 | 0.14 |
| LR chi^2^ | 210.30 | | | |
| Prob > chi^2^ | <0.001 | | | |
| Pseudo R^2^ | 0.059 | | | |
| *T5: follow-up at home 12 months after admission to rehabilitation centre, CI confidence interval* | | | | |
